# Taxonomy and Phylogeny of Stipitate Stereoid Basidiomycetes from China

**DOI:** 10.3390/jof12060400

**Published:** 2026-05-31

**Authors:** Jia-Xue Liu, Lin-Jiang Zhou, Ya-Quan Zhu, Hyang Burm Lee, Hai-Sheng Yuan

**Affiliations:** 1 CAS Key Laboratory of Forest Ecology and Silviculture, Institute of Applied Ecology, Chinese Academy of Sciences, Shenyang 110016, China; liujiaxue5t5@163.com (J.-X.L.); zhoulj24@163.com (L.-J.Z.); 14740578353@163.com (Y.-Q.Z.); 2 University of Chinese Academy of Sciences, Beijing 101408, China; 3 Environmental Microbiology Lab, Department of Agricultural Biological Chemistry, College of Agriculture & Life Sciences, Chonnam National University, Gwangju 61186, Republic of Korea; hblee@chonnam.ac.kr

**Keywords:** *Podoscypha*, *Cymatoderma*, *Stereopsis*, ITS and nLSU, new taxa

## Abstract

Stipitate stereoid fungi are saprotrophic basidiomycetes characterized by a leathery basidiome, a central-to-lateral stipe and infundibuliform pilei. Although numerous species of stipitate stereoid fungi have been recorded worldwide, understanding of their phylogenetic relationships remains extremely limited, and research on this group of fungi in China is insufficient. In this study, specimens of the three stipitate stereoid genera, namely *Podoscypha* s. l., *Cymatoderma* s. l. and *Stereopsis* s. l., from southern China were investigated. Phylogenetic analyses of the internal transcribed spacer (ITS) regions and the large subunit of the nuclear ribosomal RNA gene (LSU) using maximum likelihood (ML) and Bayesian inference (BI) methods revealed that all three genera are polyphyletic. Consequently, *Podoscypha* s. s. and *Cymatoderma* s. s. were delimited, and *Cladoderris*—previously synonymized with *Cymatoderma*—was resurrected. *Cladoderris* is characterized by an imbricate basidiome, tomentose pilei and basidiospores typically shorter than 4 μm in length. Three new species, *Podoscypha casiae*, *Stereopsis buccinata* and *Cladoderris perennis*, were described and illustrated. The morphological distinctions and affinities between the new species and closely related taxa were discussed, the thresholds for the intraspecific and interspecific demarcation within the three genera in this study were provided, and identification keys for the species of each genus were presented.

## 1. Introduction

Stipitate stereoid fungi are a group of basidiomycetes characterized by the presence of stipes, infundibuliform pilei, a smooth hymenial surface, and smooth basidiospores [1]. This group includes *Podoscypha* Pat., *Cymatoderma* Jungh., *Cotylidia* P. Karst., and *Stereopsis* D.A. Reid [1,2,3]. These fungi have a global distribution, ranging from tropical to boreal zones, and typically inhabit fallen trunks, stumps and other lignocellulose debris or grow on the ground. As decomposers, they facilitate material and nutrient cycling and play important roles in terrestrial ecosystems.

The genus *Podoscypha*, with its type species *Podoscypha nitidula* (Berk.) Pat., was first named and described in 1900 by the French mycologist Pierre Antoine Patouillard [4]. The genus is widely distributed throughout the tropical and temperate regions globally, usually growing on fallen wood or on the ground. In early taxonomic studies, species of this genus were initially classified in the family Polyporaceae, subsequently transferred to Thelephoraceae, and ultimately placed in Podoscyphaceae (Polyporales) based on molecular phylogeny [5,6,7,8,9,10]. According to MycoBank (http://www.MycoBank.org, accessed on 1 May 2026) and Index Fungorum (http://www.indexfungorum.org, accessed on 1 May 2026), the genus comprises 57 legitimate species, and 17 *Podoscypha* species have been reported or described from China [10,11,12,13,14,15,16,17]. Among them, *P. densidisca*, *P. guangxiensis*, *P. infundibula*, *P. lactea*, *P. petalodes* subsp. *cystidiata*, *P. tropica*, and *P. yunnanensis* have been discovered and identified as new species in Guangxi and Yunnan provinces, China [10,13,15].

*Cladoderris* was established by Persoon in 1826 [18], with *C. dendritica* as the type. Junghuhn established *Cymatoderma* in 1840 [19], but Fries [20] later adopted *Cladoderris*, causing *Cymatoderma* to be overlooked. This resulted in the misclassification of numerous species until Reid [21,22] confirmed the priority of *Cymatoderma* and formally synonymized *Cladoderris*. With the advent of molecular identification techniques and a reevaluation of morphological characteristics, species formerly assigned to *Cladoderris* have since been transferred to *Cymatoderma*, *Stereopsis*, *Craterellus*, and other genera [2,21,23]. Nevertheless, the taxonomic placement of a small number of species remains uncertain due to the lack of molecular data. The genus *Cymatoderma* is predominantly found in tropical regions, comprising 15 legitimate species. It has not yielded any new species from China.

The genus *Stereopsis*, whose type species is *Stereopsis radicans* (Berk.) D.A. Reid, was originally described and defined in 1965 by the British mycologist David Arthur Reid [2] and is known in most tropical and subtropical regions of the world, usually growing on the ground. With the advancement of molecular systematic studies, it has been classified in the family Stereopsidaceae (Stereopsidales) [24,25]. The genus comprises 22 legitimate species [24,25,26,27], among which one novel species (*S. yunnanensis*) has been described from China [25].

The genus *Cotylidia*, typified by *Cotylidia undulata* (Fr.) P. Karst., was established in 1881 by the mycologist Petter Karsten [28] and recently classified in Rickenellaceae (Hymenochaetales) [1,29,30]. A recent phylogenetic study revealed its polyphyletic status, confirmed *Cotylidia* sensu stricto, and proposed a new genus, *Neocotylidia* Jing Si & Hai J. Li [31]. As the taxonomy of *Cotylidia* has been recently revised, it is not included in the present study.

In recent decades, research on wood-inhabiting fungi in China has made significant progress [32,33,34], yet studies on stipitate stereoid basidiomycetes remain limited. In the present study, dozens of stipitate stereoid fungal specimens from China have been collected, and several undescribed taxa have been identified based on morphological characteristics and phylogenetic analysis of ITS and LSU rDNA sequences. The objectives of this study are to describe the new taxa of stipitate stereoid basidiomycetes from China and to clarify the taxonomic status of *Podoscypha* and *Cymatoderma* as well as their intergeneric relationships using multi-gene phylogenetic analyses.

## 2. Materials and Methods

### 2.1. Specimen Collection and Morphological Studies

Specimens were collected from Guangzhou South China National Botanical Garden (113°22′2″ E, 23°10′54″ N, altitude: 20–327 m), Shenzhen Fairy Lake Botanical Garden (114°10′32″ E, 22°34′56″ N, altitude: 60–944 m), and Shenzhen Wutong Mountain (114°13′11″ E, 22°35′05″ N, altitude: 692–944 m), Guangdong Province, South China. The regions belong to the South Asian tropical monsoon climate zone. The fresh fruiting bodies of the fungi were dried using an electronic dryer (Evermat, Bjurholm, Sweden) set at temperatures of 40–50 °C. After drying, they were labeled and stored in plastic bags and envelopes. The dried materials studied in this paper are deposited in the herbarium of the Institute of Applied Ecology, Chinese Academy of Sciences (IFP).

The macro-morphological features of the fungal specimens were observed either directly or using a stereomicroscope (Nikon SMZ 1000, Tokyo, Japan). Color terms were defined based on the standards provided by Rayner [35] and Munsell [36]. Micro-morphological data were obtained from dried specimens and observed under a light microscope (Nikon Eclipse E600 microscope, Tokyo, Japan) following the methods described by Mu et al. [37]. Samples for microscopic examination were individually mounted in cotton blue (OriLeaf, Shanghai, China), Melzer’s reagent (OriLeaf, Shanghai, China), and 5% potassium hydroxide (KOH, Damao, Tianjin, China). The range of basidiospores provided in the study excludes the smallest and largest 5% of measurements, denoted in parentheses. The following abbreviations are used in this study: KOH = 5% potassium hydroxide water solution, CB = cotton blue, CB+ = cyanophilous, CB− = acyanophilous, IKI = Melzer’s reagent, IKI− = both inamyloid and indextrinoid, L = mean spore length, W = mean spore width, Q = the range of length-to-width ratios of the basidiospores studied, Qm = mean length-to-width ratios of the basidiospores studied and n for the total number of basidiospores.

### 2.2. Molecular Analysis

#### 2.2.1. DNA Extraction and PCR

Genomic DNA was extracted from the fungal fruiting bodies using the Rapid Fungi Genomic DNA Isolation Kit, produced by Demeter Biotech Ltd., Beijing, China. The extracted DNA was subsequently used in polymerase chain reaction (PCR) procedures. The PCR amplification system is shown in Table 1. The 2× Taq PCR Mix was purchased from Tiangen Biotech (Beijing) Co., Ltd., Beijing, China.

Two molecular markers were amplified by PCR: the internal transcribed spacer (ITS) region of nuclear ribosomal DNA and the partial large subunit (nLSU) of the nuclear ribosomal RNA gene. The primer pairs used for amplification are listed as follows:

ITS region: ITS1 (5′-CTTGGTCATTTAGAGGAAGTAA-3′) and ITS4 (5′-TCCTCCGCTTATTGATATGC-3′) [38];

nLSU region: LROR (5′-ACCCGCTGAACTTAAGC-3′) and LR7 (5′-TACTACCACCAAGATCT-3′) [39].

The PCR amplification programs for the two markers were set as follows:

ITS region: Initial denaturation at 95 °C for 3 min, followed by 34 cycles of denaturation at 95 °C for 30 s, annealing at 58 °C for 30 s, extension at 72 °C for 60 s, final extension at 72 °C for 5 min, and holding at 14 °C.

LSU region: Initial denaturation at 95 °C for 3 min, followed by 39 cycles of denaturation at 95 °C for 30 s, annealing at 47.2 °C for 30 s, extension at 72 °C for 60 s, final extension at 72 °C for 5 min, and holding at 14 °C.

#### 2.2.2. Sequencing and Sequence Assembly

DNA sequencing was performed at the Beijing Genomics Institute (BGI), and all sequences generated in this study have been submitted to GenBank. The newly obtained sequences were then compared against those in the NCBI GenBank database using the BLAST+ 2.16.0 search tool (https://blast.ncbi.nlm.nih.gov, accessed on 27 September 2024) [40]. Sequence alignments were performed using ClustalX 1.8 [41]. The alignments were manually adjusted to achieve optimal alignment and minimal gaps. Sequence identity and similarity calculations were performed using BioEdit 5.0.6 software [42]. The additional ITS rDNA and nLSU rDNA sequences included in the dataset, which were used to infer phylogenetic relationships, were retrieved from GenBank (http://www.ncbi.nlm.nih.gov/genbank, accessed on 6 August 2025), as detailed in Table 2.

### 2.3. Phylogenetic Analyses

Phylogenetic relationships were inferred using both maximum likelihood (ML) and Bayesian inference (BI) analyses. The ML analysis was conducted using RAXMLGUI 2.0 [67,68]. The analysis utilized the rapid bootstrap algorithm and the GTR + I + G4 model of nucleotide substitution with 1000 bootstrap replicates.

BI analysis was performed using MrBayes 3.2.7 [69]. The nucleotide substitution model was determined as GTR + I + G based on the Bayesian information criterion (BIC) (Nst = 6, Rates = Invgamma). MCMC analysis was run for 10,000,000 generations, sampling every 4000 generations and printing trees/log-likelihood values every 1000 generations. Two independent runs were performed, each with eight chains. After discarding the initial 25% of trees as burn-in, posterior probabilities were estimated from the remaining trees to assess nodal support. Convergence of the two independent runs was assessed by the average standard deviation of split frequencies (ASDSF), with a value below 0.05 considered indicative of adequate convergence.

### 2.4. Genetic Distance Analysis and Species Delimitation Method

The genetic distance analysis was conducted in MEGA11 [70]. Specifically, the following parameters were used: the Kimura 2-parameter model was selected as the substitution model [71], and the bootstrap method was set to 10,000 replicates. In addition, rates among sites were assumed to be uniform. Pairwise deletion was applied for missing data treatment.

The visualization of genetic distance analysis was performed in R 4.5.2, utilizing the pheatmap and corrplot packages [72,73,74].

To assess species boundaries within key taxa, we performed Bayesian Poisson Tree Processes (bPTP) analysis following the method of Zhang et al. [75], using the web server (https://species.h-its.org/ptp/, accessed on 1 May 2026). The input phylogenetic tree was the maximum likelihood (ML) tree inferred from the concatenated ITS + nLSU dataset. Analyses were conducted with the following parameters: the tree was set as rooted; the Markov chain Monte Carlo (MCMC) was run for 250,000 generations, with a thinning interval of 100 and a burn-in of 0.15. The “remove outgroup” option was enabled to improve delimitation performance. MCMC convergence was assessed by examining the trace of log-likelihood values. The resulting species delimitation posterior probabilities (PPs) were visualized on the phylogenetic tree to identify putative species-level lineages. According to Malavasi et al. [76], clades with posterior probability (PP) ≥ 0.95 were considered highly well supported.

## 3. Results

### 3.1. Molecular Phylogeny

#### 3.1.1. Podoscypha, Cymatoderma and Cladoderris

**Figure 1:**
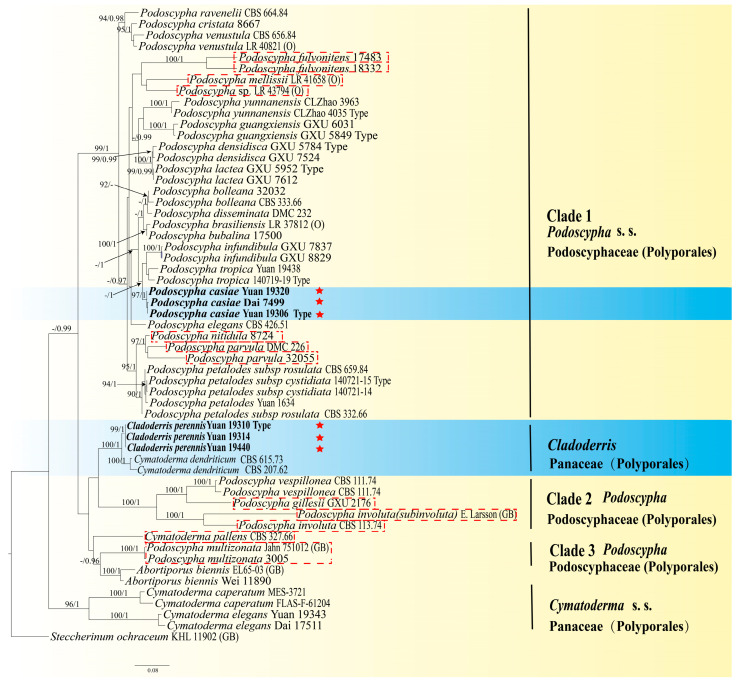
ML phylogenetic tree of the genera *Podoscypha*, *Cymatoderma*, and *Cladoderris* based on ITS + nLSU. Branches are labeled with maximum likelihood bootstrap values (≥80%) and Bayesian posterior probabilities (≥0.95). Red dotted lines indicate species units supported by bPTP (PP ≥ 0.95). The two new species are shown in bold and marked with red stars. All new taxa and the resurrected genus are highlighted within the blue area.

The dataset of *Podoscypha*, *Cymatoderma*, and *Cladoderris*, based on ITS + nLSU regions, comprises 57 samples, with *Steccherinum ochraceum* (Pers. ex J.F. Gmel.) Gray designated as the outgroup. The data matrix consists of 1373 constant characters, 318 parsimony-uninformative variable characters, and 509 parsimony-informative sites, resulting in an alignment length of 2200 bases. Both ML and BI analyses were applied to this identical dataset and aligned sequences, generating phylogenetic trees with similar topological structures, as depicted in Figure 1.

In this study (Figure 1), most species of *Podoscypha* form a well-supported clade (99% ML, 1.00 BPP) together with the type species of the genus, *Podoscypha nitidula*; this clade is recognized as *Podoscypha* sensu stricto. *Podoscypha gillesii* Boidin & Lanq., *P. involuta* (Klotzsch ex Fr.) Imazeki, *P. subinvoluta* Jing Si & Hai J. Li, and *P. vespillonea* (Berk.) Boidin & Lanq. form a distinct clade with full support (100% ML, 1.00 BPP). Due to the unavailability of these specimens, they are provisionally retained in *Podoscypha*. Three specimens of *Podoscypha casiae* J.X. Liu & H.S. Yuan (Yuan 19306, Yuan 19320 and Dai 7499) form a highly supported clade (97% ML, 1.00 BPP) and cluster with the clade containing *P. tropica* Jing Si & Hai J. Li and *P. infundibula* F.C. Huang & H.F. Zheng with moderate support (1.00 BPP).

In the phylogenetic tree (Figure 1), the type species of the genus, *Cymatoderma elegans*, and *C. caperatum* (Berk. & Mont.) D.A. Reid form a highly supported clade (96% ML, 1.00 BPP), which is regarded as *Cymatoderma* sensu stricto. *Cymatoderma dendriticum* (Pers.) D.A. Reid and three undescribed samples (Yuan 19310, Yuan 19314 and Yuan 19440) form a fully supported clade (100% ML, 1.00 BPP), prompting the resurrection of the genus *Cladoderris* to accommodate this lineage.

The bPTP species delimitation analysis showed good MCMC convergence, confirming reliable results. The bPTP analysis based on the ML tree delimited a total of 27 putative species (additional Supporting Information in Supplementary Material S1). Among these, 12 species received relatively high delimitation support (PP ≥ 0.95), including *Podoscypha nitidula*, *P. mellissii*, *P. gillesii* and *Cymatoderma pallens*, supporting their recognition as independent species. However, sequences from different specimens of the same species, such as *P. fulvonitens*, *P. parvula*, and *P. involuta*, were also split into distinct species units, indicating potential over-splitting by bPTP. By contrast, the PP values of the *P. casiae* species complex (including *P. bolleana*, *P. brasiliensis*, *P. bubalina*, *P. disseminata*, *P. tropica*, and *P. infundibula*), the *C. dendriticum*–*Cladoderris perennis* clade, and *C. caperatum* ranged from 0.40 to 0.60, indicating low delimitation confidence and ambiguous species boundaries.

#### 3.1.2. Stereopsis

**Figure 2:**
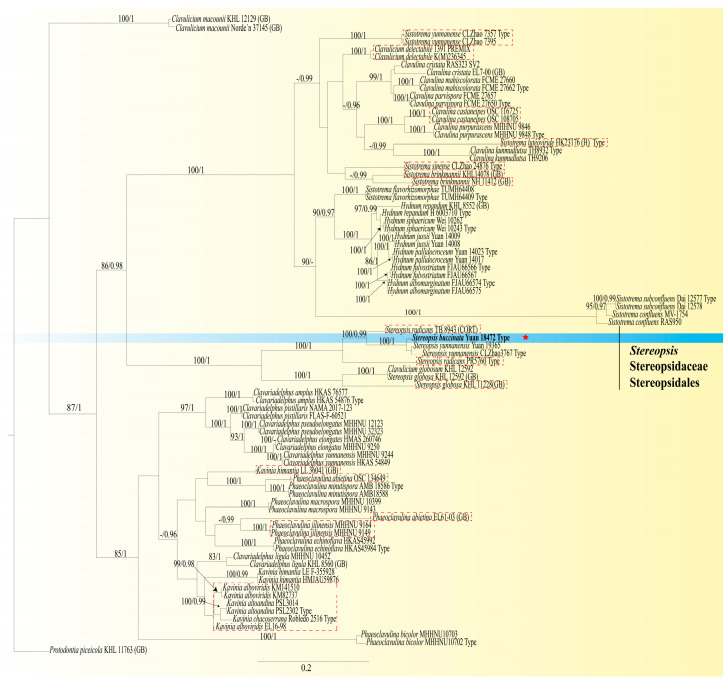
ML phylogenetic tree of the genus *Stereopsis* based on ITS + nLSU. Branches are labeled with maximum likelihood bootstrap values (≥80%) and Bayesian posterior probabilities (≥0.95). Red dotted lines indicate species units supported by bPTP (PP ≥ 0.95). The one new species is in bold and marked with a red star. The new taxa is highlighted within the blue area.

The ITS + nLSU dataset for *Stereopsis* includes 82 samples, with *Protodontia piceicola* (Kühner ex Bourdot) G.W. Martin designated as the outgroup. The data matrix comprises 903 constant characters, 124 parsimony-uninformative variable characters, and 1046 parsimony-informative sites, with an alignment length of 2073 bases. Both ML and BI analyses of this dataset generated phylogenetic trees with similar topologies, as shown in Figure 2.

In the phylogenetic tree (Figure 2), *Stereopsis yunnanensis* Qi Yuan & C.L. Zhao cluster with *Stereopsis buccinata* J.X. Liu & H.S. Yuan to form a clade with full support (100% ML, 1.00 BPP). *Stereopsis globosa* (Hjortstam & Ryvarden) Sjökvist, *S. radicans*, *S. yunnanensis*, and *S. buccinata* formed a fully supported clade (100% ML, 1.00 BPP).

The bPTP species delimitation analysis showed good MCMC convergence, confirming reliable results. Species delimitation analysis using bPTP based on the maximum likelihood (ML) phylogenetic tree recovered a total of 39 putative species (additional Supporting Information in Supplementary Material S2). Among these, 15 species received relatively high delimitation support (PP ≥ 0.95); these include *Clavulicium delectabile*, *Sistotrema brinkmannii*, *S. globosa*, *S. radicans*, and *Kavinia himantia*. In addition, *S. yunnanensis* and *S. buccinata* were delimited as a single species unit with a PP value of 0.90.

### 3.2. Genetic Distance Analysis

**Figure 3:**
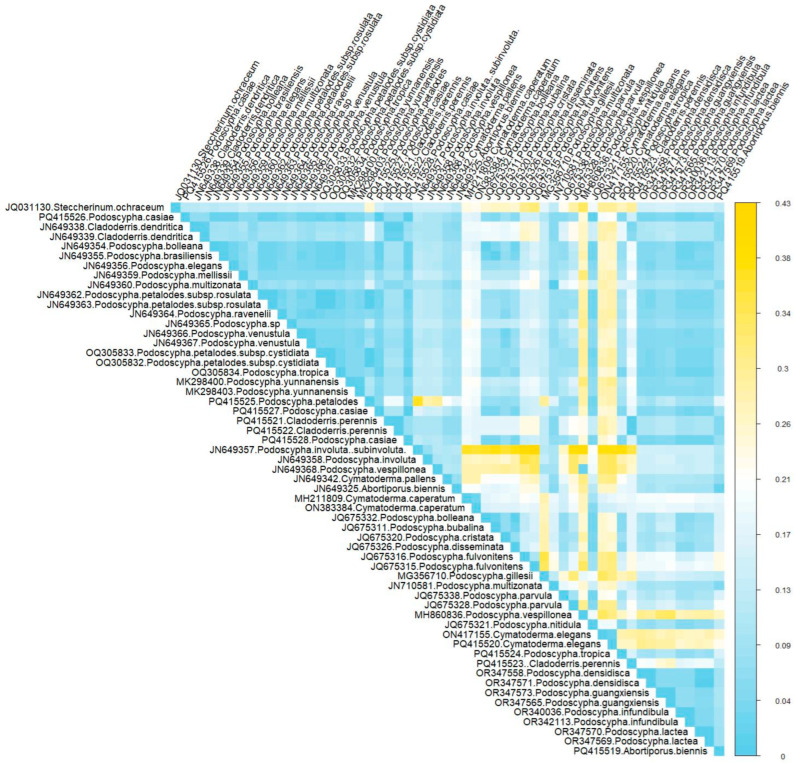
Genetic distance heatmap for *Podoscypha*, *Cymatoderma*, and *Cladoderris* based on ITS + nLSU sequences (Kimura 2-parameter model). Colors indicate pairwise genetic distances, ranging from low (blue) to high (yellow), with larger values representing greater sequence divergence and more distant phylogenetic relationships.

Genetic distances among 57 concatenated ITS and nLSU sequences were calculated under the Kimura 2-parameter model in MEGA 11 and visualized as a heatmap (Figure 3; full matrix in Supplementary Material S3). Distances range from 0 (identical) to 0.43 (highly divergent), with the color gradient transitioning from white (distance < 0.04) to sky blue (>0.30).

As shown in the genetic distance heatmap (Figure 3), interspecific genetic distances within *Podoscypha* s. s. range from 0.02 to 0.22. In *Cymatoderma* s. s., interspecific distances are relatively high (0.145–0.313), while intraspecific distances are low (0.024–0.03). Distances between *Cymatoderma* s. s. and *C. pallens* range from 0.277 to 0.313. By contrast, interspecific distances within *Cladoderris* are small (0.015–0.052), with intraspecific distances ranging from 0 to 0.01. Intergeneric distances are as follows: between *Podoscypha* s. l. and *Cladoderris*, 0.05–0.353; between *Podoscypha* s. s. and *Cladoderris*, 0.063–0.257; between *Podoscypha* s. l. and *Cymatoderma*, 0.048–0.38; between *Podoscypha* s. s. and *Cymatoderma* s. s., 0.048–0.317; and between *Cymatoderma* s. s. and *Cladoderris*, 0.175–0.282. Hence, the minimum intraspecific/interspecific genetic distances for *Podoscypha*, *Cymatoderma*, and *Cladoderris* were 0/0.006, 0.024/0.145, and 0/0.015, respectively. The minimum intergeneric distances between *Podoscypha* s.s. and the latter two genera were 0.048 and 0.063, respectively, while that between *Cymatoderma* and *Cladoderris* was 0.061. In addition, the minimum intergeneric distance between clades 2 and 3 of *Podoscypha* and *Cladoderris* was 0.05.

### 3.3. Taxonomy

#### 3.3.1. Delimitation of the Genera

Based on the results of phylogenetic analysis (Figure 1 and Figure 2), the generic characteristics of *Podoscypha* s. s. and *Cymatoderm* s. s. were redefined.

***Podoscypha*** Pat., Essai Tax. Hyménomyc. (Lons-le-Saunier): 70 (1900).

**Type species.** *Podoscypha nitidula*.

**Description.** Basidiome flabellate to infundibuliform, with stipe length exhibiting considerable variation; pileal surface smooth or tomentose, featuring radiating wrinkles or ridges; hymenial surface smooth or with radiating stripes; hyphal structure dimitic, comprising generative hyphae with clamp connections and thick-walled skeletal hyphae; cystidia present; basidia clavate, with a basal clamp; basidiospores ellipsoid to cylindrical, smooth, thin-walled, hyaline, and typically exceeding 3 µm in length.

**Distribution.** Most of the world except for Northern Asia, Central Asia, and the polar regions.

**Substrate.** Ground or fallen wood.

**Notes.** *Podoscypha*, first described and established in 1900, is a genus within the family Podoscyphaceae. Globally, 57 species of this genus have been recognized to date, predominantly distributed across tropical and temperate regions. In China, 17 species have been recorded, primarily inhabiting tropical to temperate areas.

***Cymatoderma*** Jungh., Tijdschr. Nat. Gesch. Physiol. 7: 290 (1840).

**Type species.** *Cymatoderma elegans*.

**Description.** Basidiome flabellate, pseudo-infundibuliform to infundibuliform, stipitate; pileal surface covered with radiating ridges; hymenial surface with radiating folds or ridges; stipe tomentose; hyphal structure dimitic, hymenium thickened; cuticular layer present, giving rise to the pileal surface tomentum, the hairs that form the tomentum thick-walled except for apices, which may be thin-walled; cystidia present; basidia clavate, with a basal clamp; basidiospores cylindrical to ellipsoid, usually longer than 5 µm.

**Distribution.** North and South America, Africa, Southeast Asia, and Oceania.

**Substrate.** Fallen wood.

**Notes.** The genus *Cymatoderma* was first described in 1840 and currently belongs to Panaceae. To date, 15 species of this genus are recognized all over the world, predominantly in tropical regions. In China, there are two species, primarily distributed in tropical and subtropical regions.

#### 3.3.2. Resurrected Genus

***Cladoderris*** (Pers.) Berk., 1842, gen. resurr.

**Etymology.** *Cladoderris* (Lat.): referring to the dendroid, leathery basidiome.

**Type species.** *Cladoderris dendritica* (Pers.) Berk., automatically reinstated.

**Description.** Basidiome flabellate to infundibuliform, imbricate, stipitate; pileal surface covered by a highly developed felt-like tomentum with indistinct blade ridges beneath it; hymenial surface exhibits prominent ridges; stipe tomentose; hyphal structure dimitic or trimitic; cuticular layer absent; cystidia present; gloeocystidia thin-walled, forming long and undulating bodies; basidia clavate to subcylindrical; basidiospores ellipsoid to subglobose.

**Distribution.** South America and Africa.

**Substrate.** Ground or fallen wood.

**Notes.** Phylogenetic analysis places *Cladoderris perennis* and *Cymatoderma dendriticum* within the same clade, distinct from the clade of *Cymatoderma* s. s. Morphologically, *Cymatoderma* s. s. and *Cladoderris* share common traits in having a flabellate to infundibuliform basidiome, a pileus surface with ridges, tomentose stipes, and the presence of cystidia, clavate basidia, and subcylindrical-to-ellipsoidal basidiospores. However, the main difference between *Cladoderris* and *Cymatoderma* lies in that *Cladoderris* species have an imbricate basidiome, lack the cuticular layer, and have basidiospores shorter than 4 μm [2].

***Cladoderris dendritica*** (Pers.) Berk.

**Basionym.**

*Thelephora dendritica* Pers., Botanique [5]: 176 (1827).

*Cymatoderma dendriticum* (Pers.) D.A. Reid, Kew Bull. [13] (3): 523 (1959).

**Notes.** The holotype of *Cladoderris dendritica* was collected from Peninsular Malaysia. It is characterized by a lateral stipe, a trimitic hyphal structure, absence of a cuticular layer, absence of cystidia, and broadly ellipsoid-to-subglobose basidiospores (3–4 × 2.5–3 μm) [2].

#### 3.3.3. New Taxa

**Figure 4:**
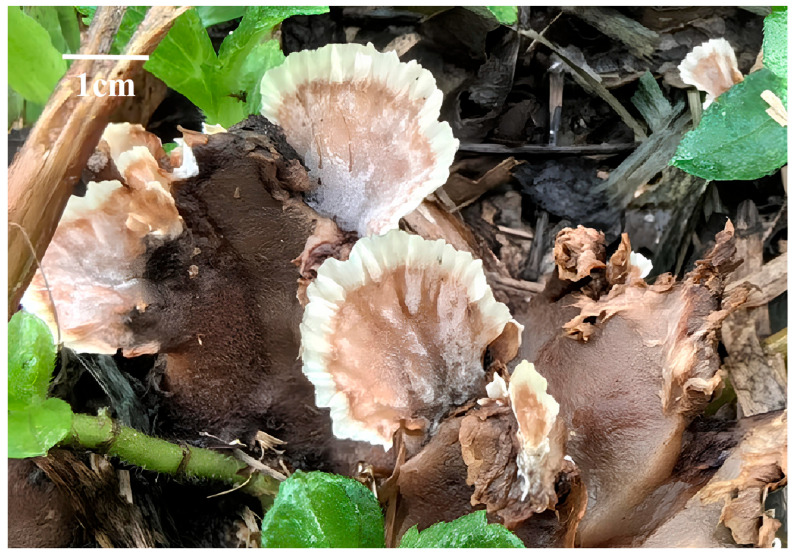
Basidiome of *Cladoderris perennis* (holotype Yuan 19310, photo by Yong-Mei Cheng).

**Figure 5:**
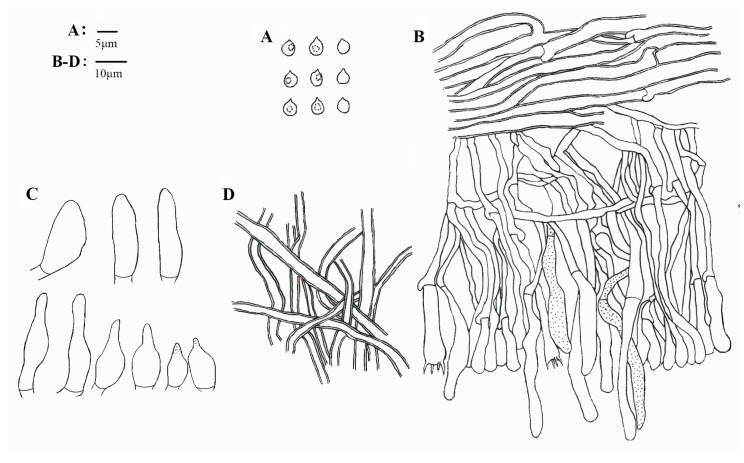
Microscopic structures of *Cladoderris perennis* (holotype Yuan 19310). (**A**) Basidiospores; (**B**) a section of hymenophore with gloeocystidia (granular) and leptocystidia (hyaline); (**C**) leptocystidia and cystidioles; (**D**) skeletal hyphae in trama. Scale bars: 5 μm (**A**); 10 μm (**B**–**D**).

***Cladoderris perennis*** J.X. Liu & H.S. Yuan, sp. nov. (Figure 4 and Figure 5).

**Fungal Names number.** FN 572903.

**GenBank accession numbers.** PQ415521 (ITS) and PQ415509 (nLSU).

**Etymology.** *Perennis* (Lat.): referring to perennial habits.

**Diagnosis.** Similar to *Cladoderris dendritica* but differs in having a dimitic hyphal system, thick-walled skeletal hyphae, and the presence of cystidia.

**Description.** Basidiome annual to perennial, stipitate, without taste, umami smell, becoming corky upon drying, 40–90 mm high. Pileus flabellate to reniform, imbricate, projecting up to 7–18 mm, 8–28 mm wide, 0.4–0.9 mm thick at the center, with a thin margin, extending towards the stipe and becoming fleshy; pileal surface golden blonde (5C4), topaz (5C5) and oak brown (5D6) when dry, smooth, with unobvious longitudinal ridges and a developed penniform tomentum; margin of pileal surface dark brown (7F6-7F8), indented. Hymenial surface orange white (5A2) to orange gray (5B2 to 5B4), tomentose, with prominent ridges under the tomentum. Absence of cuticular layer. Stipe lateral or central, soft corky, inflated, great variation in length, up to 70 mm long, sand (4B3) and pale orange (5A3), with yellowish white tomentum. Hyphal system dimitic; generative hyphae with clamps, thin- to slightly thick-walled; skeletal hyphae thick-walled, amyloid in IKI, CB+; tissues unchanged in KOH. Generative hyphae in context with clamps, colorless, thick-walled, frequently branched, subparallel to slightly interwoven, 2.1–5 μm in diam; skeletal hyphae in trama colorless, thick-walled to almost solid, unbranched, 2.8–5.3 μm in diam. Cystidia (leptocystidia) pyriform, clavate to subcylindrical, without apex, frequently present, 18–58 × 2–11 μm, sometimes encrusted; pilo- and caulocystidia absent. Gloeocystidia abundant, thin-walled, forming long and undulating bodies, 32–48 × 3.5–6 μm. Basidia narrowly clavate to subcylindrical, with four sterigmata and a basal clamp, 30–38 × 4.2–6.7 μm; basidioles dominant, in shape similar to basidia, clamps, slightly smaller, thin-walled. Basidiospores subglobose to ellipsoid, colorless, smooth, often monoguttulate, thin-walled, IKI–, CB–, 3.3 ± 0.2 × 3.0 ± 0.1 μm (2.9–4.2 × 2.7–3.2), L = 3.3 μm, W = 3.0 μm, Q = 1.1–1.3, Qm = 1.1, n = 90.

**Distribution.** Currently known only from China.

**Substrate.** On the ground.

**Holotype.** China, Guangdong Province, Shenzhen City, on the ground, 24 Apr. 2024, collected by Yong-Mei Cheng (holotype designated IFP 019974, specimen Yuan 19310).

**Additional specimens examined (paratypes).** China, Guangdong Province, Shenzhen City, on the ground, 26 April 2024, collected by Li-Fang Peng (paratype designated IFP 019975, specimen Yuan 19314); on the ground, 21 June 2024, collected by Yong-Mei Cheng (paratype designated IFP 019976, specimen Yuan 19440).

**Notes.** The new species is characterized by the absence of a cuticular layer, an inflated stipe and the presence of cystidia. *Cladoderris perennis* and *C*. *dendritica* form a sister clade, which share robust stipes and tomentose pilei with prominent ridges. The differences between *C*. *dendritica* and *C*. *perennis* are that *C. perennis* has a dimitic hyphal system, thick-walled skeletal hyphae, and the presence of cystidia, whereas *C. dendritica* possesses a trimitic hyphal system with thin-walled skeletal hyphae and lacks cystidia [2]. Furthermore, the pairwise ITS sequence similarity value between the new species (Specimen Yuan 19310) and *C. dendritica* (Specimen CBS 207.62) was 95.3%.

**Figure 6:**
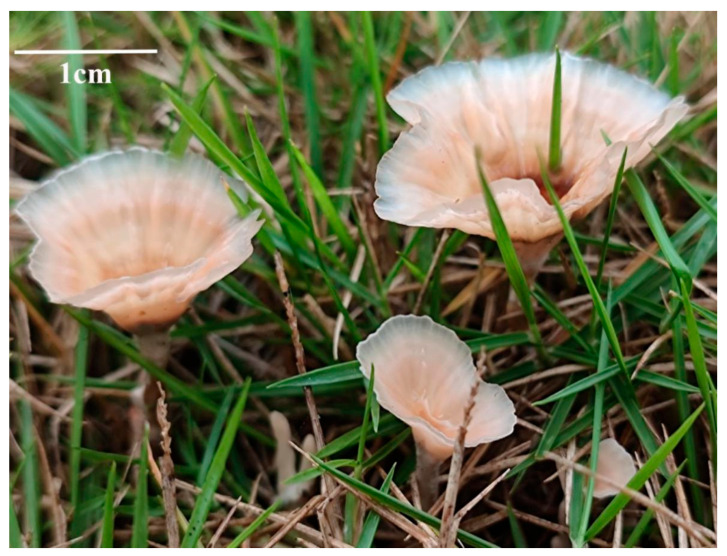
Basidiome of *Podoscypha casiae* (holotype Yuan 19306, photo by Zhen-Xing Yao).

**Figure 7:**
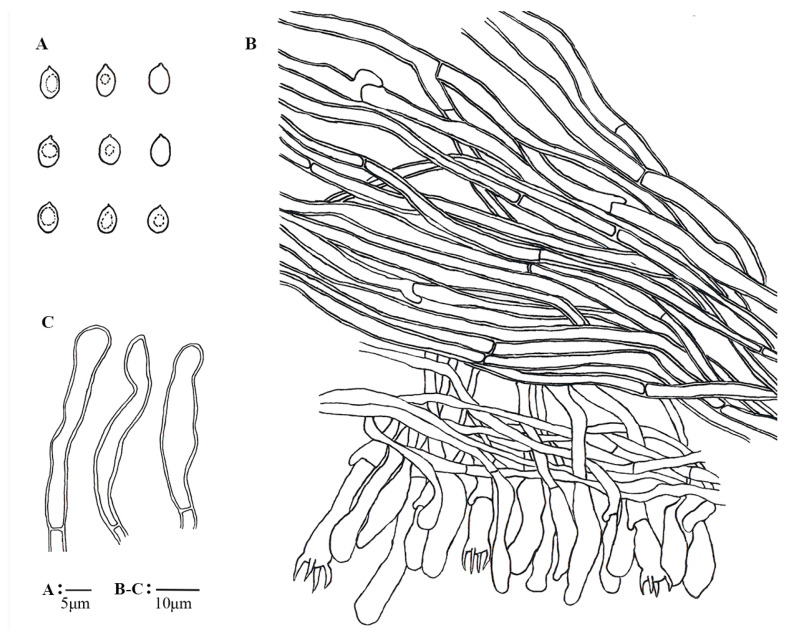
Microscopic structures of *Podoscypha casiae* (holotype Yuan 19306). (**A**) Basidiospores; (**B**) a section of hymenophore with basidia and cystidia; (**C**) caulocystidia. Scale bars: 5 μm (**A**); 10 μm (**B**,**C**).

***Podoscypha casiae*** J.X. Liu & H.S. Yuan, sp. nov. (Figure 6 and Figure 7).

**Fungal Names number.** FN 572901.

**GenBank accession numbers.** PQ415526 (ITS) and PQ415513 (nLSU).

**Etymology.** *Casiae* (combination of CAS and IAE), commemorating the 70th anniversary of the Institute of Applied Ecology (IAE), Chinese Academy of Sciences (CAS).

**Diagnosis.** Similar to *Podoscypha tropica* but differs in tomentose hymenial surface and CB+ skeletal hyphae.

**Description.** Basidiome annual, stipitate, gregarious, without odor or taste, coriaceous when fresh, becoming hard upon drying, 35–70 mm high. Pileus pseudoinfundibuliform to infundibuliform, projecting up to 13–18 mm long, 10–13 mm wide, 0.2–0.3 mm thick at the center; pileal surface of fresh specimens orange white (5A2) and pale red (7A3), light brown (6D8) when dry, glabrous, with distinct longitudinal stripe and rust brown concentric zones; margin of pileal surface Pompeian yellow (5C6) and clay (5D5) when dry, entire or slightly dentiform. Hymenial surface concolor or paler with pileal surface, very minutely tomentose. Stipe central, slender, cylindrical, up to 50 mm long and 3 mm in diam, golden brown to light brown (5D7–5D8) when dry, with grayish-white tomentum. Hyphal system dimitic; generative hyphae with clamps, thin- to thick-walled; skeletal hyphae thick-walled; IKI–, CB+, tissues unchanged in KOH. Generative hyphae in context dominant, with clamps and simple septs, colorless, thick-walled, moderately branched, subparallel to loosely interwoven, 2.3–3.9 μm in diam; skeletal hyphae in context colorless, thick-walled, unbranched, 3.1–5.4 μm in diam. Cystidia cylindrical, which has no apex, rarely present, thin-walled, 19–34 × 5.2–5.7 μm; pilocystidia absent; caulocystidia present, clavate to subcylindrical, thick-walled, 56–61 × 8.9–10 μm; basidia narrowly clavate to subcylindrical, with four sterigmata and a basal clamp, 9.7–28.5 × 4.0–4.6 μm; basidioles dominant, in shape similar to basidia, clamps, slightly smaller, thin-walled. Basidiospores ellipsoid to broadly ellipsoid, colorless, smooth, often monoguttulate, thin-walled, IKI–, CB–, 4.9 ± 0.67 × 3.3 ± 0.55 μm (4.0–7.0 × 2.4–4.3), L = 4.9 μm, W = 3.3 μm, Q = 1.3–2.0, Qm = 1.5, n = 90.

**Distribution.** Currently known only from China.

**Substrate.** On buried wood.

**Holotype.** China, Guangdong Province, Shenzhen City, on buried wood, 22 Apr. 2024, collected by Zhen-Xing Yao (holotype designated IFP 019977, specimen Yuan 19306).

**Additional specimens examined (paratypes).** China, Guangdong Province, Shenzhen City, on buried angiosperm wood, 24 May 2006, collected by Yu-Cheng Dai (paratype designated IFP 011924, specimen Dai 7499); on fallen angiosperm trunk, 26 April 2024, collected by Jian-Feng Tan (paratype designated IFP 019978, specimen Yuan 19320).

**Notes.** The new species is characterized by a glabrous pileal surface, a minutely tomentose hymenial surface and the presence of stipe and cystidia. *Podoscypha casiae* closely resembles *P. tropica* and *P. infundibula* in having caulocystidia and ellipsoid basidiospores. However, *P*. *casiae* differs from *P. tropica* in its tomentose hymenial surface and stipe, thin- to thick-walled generative hyphae, and CB+ skeletal hyphae [15]. *P*. *casiae* can be distinguished from *P. infundibula* in its smooth pileal surface and larger basidiospores (4.9 ± 0.67 × 3.3 ± 0.55 μm) [13]. Moreover, pairwise ITS sequence similarity analysis showed that the new species (Specimen Yuan 19306) had 97.8% similarity with *P. tropica* (Specimen 140719-19).

**Figure 8:**
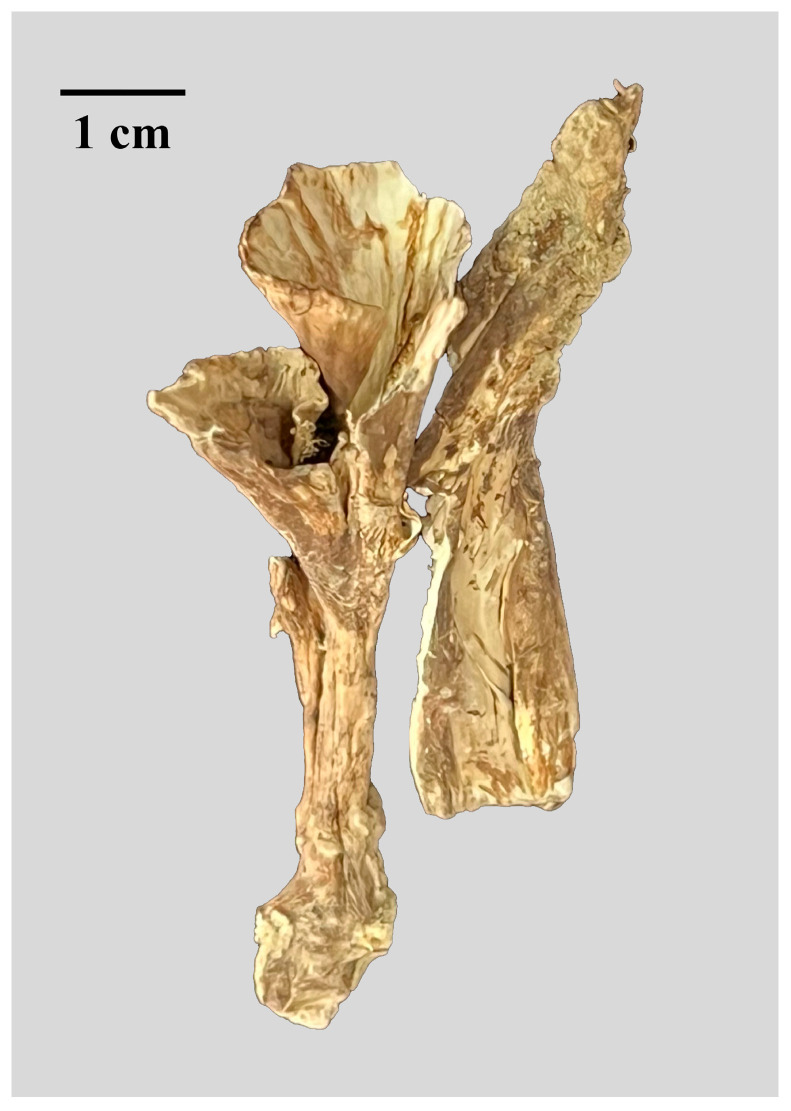
Basidiome of *Stereopsis buccinata* (holotype Yuan 18472; photo by Jia-Xue Liu).

**Figure 9:**
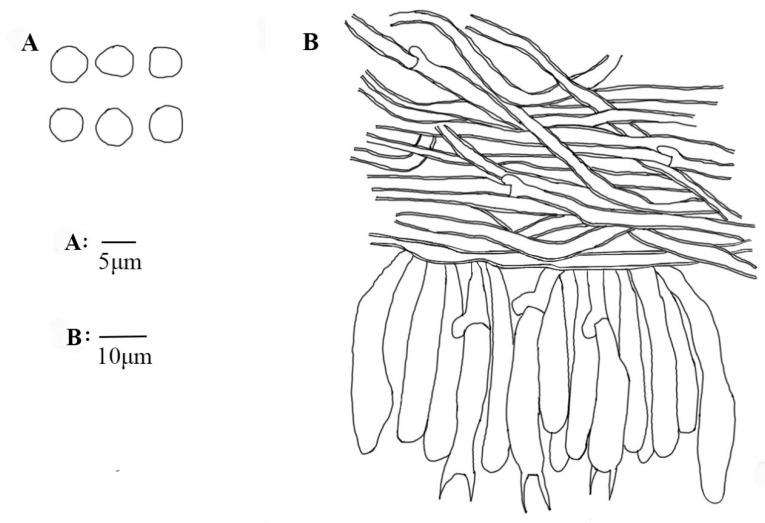
Microscopic structures of *Stereopsis buccinata* (holotype Yuan 18472). (**A**) Basidiospores; (**B**) a section of hymenophoral trama with cystidia and basidia. Scale bars: 5 μm (**A**); 10 μm (**B**).

***Stereopsis buccinata*** J.X. Liu & H.S. Yuan, sp. nov. (Figure 8 and Figure 9).

**Fungal Names number.** FN 572925.

**GenBank accession numbers.** PV190982 (ITS) and PV174382 (nLSU).

**Etymology.** *Buccinata* (Lat.): referring to the trumpet-shaped basidiome.

**Diagnosis.** Similar to *Stereopsis yunnanensis* but differs in basidiome without odor, tomentose pileal surface and hymenial surface and larger basidiospores.

**Description.** Basidiome annual, stipitate, solitary, without odor or taste, becoming hard upon drying, 12–41 mm high. Pileus flabelliform or infundibuliform, projecting up to 7–21 mm long, 9–22 mm wide, 0.2–0.4 mm thick at the center; pileal surface of dried specimens cream (4A3) or corn (4B5), tomentose, without radial stripes or zones; margin of pileal surface Somali (7E5) or eye brown (7F6), entire, wavy, with radial folds. Hymenial surface chocolate (6F4) or dark brown (8F4), with a thin and gray–white tomentum. Stipe lateral, bended, flattened, robust, up to 21 mm long and 5 mm in diam, with a white tomentum, concolor or paler with hymenial surface when dry. Hyphal system monomitic; generative hyphae slightly thick-walled, unbranched, IKI–, CB–; tissues unchanged in KOH. Generative hyphae in context colorless, subparallel or loosely interwoven, 1.5–2.2 μm in diam. Cystidia cylindrical, thin-walled, 35–47 × 3.5–5 μm; pilo- and caulocystidia absent. Basidia clavate, with two sterigmata and a basal clamp, 30–40.2 × 3.6–4 μm; basidioles dominant, similar to basidia in shape, generally slightly smaller. Basidiospores subglobose, colorless, smooth, thin-walled, IKI–, CB–, 5.9 ± 0.52 × 5.2 ± 0.45 μm (5.0–7.0 × 4.5–6.0), L = 5.9 μm, W = 5.2 μm, Q = 1.04–1.3, Qm = 1.1, n = 30.

**Distribution.** Currently known only from China.

**Substrate.** On the ground.

**Holotype.** China, Guangdong Province, Guangzhou City, Tianhe District, on the ground, 23 August 2023, collected by Hai-Sheng Yuan (holotype designated IFP 020068, specimen Yuan 18472).

**Notes.** The new species is characterized by a tomentose pileal surface, a hymenial surface, and basidia with two sterigmata. *Stereopsis buccinata* clusters with *S. yunnanensis* and *S. radicans*, displaying similarities in having a monomitic hyphal system with thick-walled generative hyphae, basidia with two sterigmata, and subglobose basidiospores. However, *S*. *buccinata* differs from *S. yunnanensis* in its basidiome without odor, tomentose pileal surface and hymenial surface, stipe with a white tomentum and larger basidiospores (5.9 ± 0.52 × 5.2 ± 0.45 μm in *S*. *buccinata* vs. 4.5–6.5 × 4–5.5 µm in *S. yunnanensis*) [25]. *Stereopsis buccinata* is distinguished from *S. radicans* by unbranched generative hyphae and smaller basidiospores (5.9 ± 0.52 × 5.2 ± 0.45 μm in *S*. *buccinata* vs. 6–8 × 5–7.5 µm in *S. radicans*) [2]. In addition, the pairwise ITS sequence similarity value between the new species and *S. yunnanensis* (Specimen CLZhao3767) was 94.7%.

## 4. Discussion

In this study, ITS and LSU regions were amplified from seven specimens of stipitate stereoid fungi collected from subtropical to temperate regions of China. We integrated phylogenetic analysis, genetic distance, bPTP, ITS sequence similarity analysis and morphology to delimit new taxa; consequently, three new species of stipitate stereoid basidiomycetes were proposed, and one genus was resurrected.

Consistent with the results of Sjökvist et al. [1], Wu et al. [10], Huang et al. [13], and Si et al. [15], the phylogenetic analyses in this study indicate that the genus *Podoscypha* is polyphyletic, forming three distinct lineages. Clade 1 contains the type species *P. nitidula* and *P. casiae*, as well as the majority of species belonging to *Podoscypha* s.s. Clades 2 and 3 include species such as *P. involuta*, *P. subinvoluta*, *P. gillesii*, *P. vespillonea*, and *P. multizonata*, all of which exhibit significant genetic differentiation in both tree topology and genetic distances. *P. casiae* and *P. tropica* exhibit distinct morphological differences, and *P. casiae* forms a highly supported monophyletic branch in the phylogenetic tree. The ITS sequence similarity between the two taxa is 97.8%, slightly above the 97% similarity cutoff. However, the bPTP species delimitation analysis provided only weak statistical support (PP 0.50 < 0.95), indicating ambiguous boundaries likely due to limited sampling or barcode resolution. Therefore, given that morphological divergence and phylogenetic independence are unequivocally supported, whereas the uncertainty in molecular evidence stems from methodological limitations, we recognize *P. casiae* as a new species based on the integration of phylogenetic and morphological evidence. Due to the limited number of specimens, five species within clades 2 and 3 are temporarily retained in the genus *Podoscypha* pending additional data.

In agreement with the findings of Yuan et al. [25], *Stereopsis radicans*, *S. yunnanensis*, and *S. globosa* cluster into a highly supported monophyletic branch. *S. buccinata* is also located within this clade and forms a well-supported sister branch with *S. yunnanensis*. The strong ITS divergence (5.3%, exceeding the 3% threshold), together with phylogenetic and morphological evidence, leads us to recognize *S. buccinata* as a new species. Although the bPTP analysis grouped *S. buccinata* and *S. yunnanensis* into a single putative species with fairly high but not significant posterior probability (PP 0.90), this value falls below our adopted significance threshold (PP ≥ 0.95), and the result is likely influenced by limited sampling or methodological limitations. Due to insufficient sequence data, the generic boundaries and internal relationships of *Stereopsis* remain unresolved. Additional specimens and molecular data are needed in the future to clarify the generic classification of this genus.

For *Cymatoderma* s.l., the phylogenetic results are consistent with those of Sjökvist et al. [1] and Huang et al. [13], showing that the genus is polyphyletic. The phylogenetic analysis supports a monophyletic clade corresponding to *Cymatoderma* s.s., which includes its type species *C. elegans*; meanwhile, *Cladoderris dendritica* and *C. perennis* form a highly supported clade. *C. pallens* clusters with *P. multizonata* and *Abortiporus biennis* (Bull.) Singer in a clade with low support. The ITS sequence similarity between *C. perennis* and *C. dendritica* is 95.3%, reflecting a divergence of 4.7%, which surpasses the 3% cutoff typically applied for species differentiation among basidiomycetes. *C. perennis* also constitutes a separate, strongly supported monophyletic clade in the phylogenetic analysis, and clear morphological distinctions provide additional evidence for its separation. Furthermore, the bPTP analysis yielded only low support (PP 0.60 < 0.95) for grouping *C. perennis* and *C. dendritica* as a single species; this outcome points to uncertain species boundaries, potentially attributable to methodological constraints or insufficient sampling. Therefore, we prioritize the robust phylogenetic independence, substantial ITS divergence, and clear morphological distinctions and designate *C. perennis* as a novel species of *Cladoderris*. Some species with no available molecular data (*C. africanum*, *C. plicatum*, *C. sclerotioides*, and *C. blumei*) are provisionally placed within *Cymatoderma* s.s. based on shared morphological features (a distinct cortex, thick-walled pileal hairs, and basidiospores ≥ 5 μm [2]).

Notably, the minimum interspecific genetic distance within *Cymatoderma* (0.145) was greater than the minimum intergeneric distances between *Cymatoderma* and both *Podoscypha* (0.048) and *Cladoderris* (0.061), which is consistent with previous studies indicating that *Cymatoderma* is not monophyletic [1,13]. However, due to the extremely limited number of *Cymatoderma* specimens and available sequences, there is insufficient evidence to reassess the taxonomic status of this genus at present. Therefore, we have followed the traditional classification scheme for *Cymatoderma* in this study and focus our discussion on the distinct generic status of *Cladoderris*. Future studies incorporating more species and population samples of *Cymatoderma* will be necessary to further test its generic boundaries.

Previous studies have placed most species of *Cladoderris* within *Cymatoderma* [21,77,78], but our results show that *Cladoderris* and *Cymatoderma* form independent, well-supported clades. The maximum interspecific genetic distance within *Cladoderris* is 0.052. With the exception of the minimum intergeneric distance between *Cladoderris* and *Podoscypha* in clades 2 and 3 (0.05), which is slightly lower than this value, the minimum intergeneric distances between *Cladoderris* and both *Podoscypha* s.s. (0.063) and *Cymatoderma* (0.061) are greater than 0.052. This slight overlap may be due to incomplete lineage sorting, limited sampling of clades 2–3, or the conserved nature of the ITS + LSU regions; nonetheless, the preponderance of evidence (monophyly, morphology, and other intergeneric distances) supports generic separation.

Stipitate stereoid fungi are widely distributed across tropical, subtropical, and temperate zones. The three new species described in this study enrich the diversity of this fungal group in China. Nevertheless, the taxonomy and distribution of related taxa in China still require further extensive sampling and investigation.

**Keys to the accepted species of *Podoscypha*, *Cymatoderma*, *Stereopsis*** **and *Cladoderris*** **worldwide**.

A key to accepted species of *Podoscypha*1. Basidiome sessile or without a distinct stipe···································································21. Basidiome with a distinct stipe·························································································72. Basidiome effused and reflexed························································································32. Stipe short and rudimentary·····························································································43. Pileocystidia present but scattered·················································*Podoscypha caespitosa*3. Pileocystidia absent·················································································*P. semiresupinata*4. Pilei surface with radial wrinkles·····················································································54. Pilei surface with zones·····································································································65. Basidia with 4 sterigmate, 43.5 × 5.5 μm···························································*P. aculeata*5. Basidia with 2 or 4 sterigmate, 18–26 × 4–6 μm·········································*P. multizonata*6. Hymenial surface ochraceous buff in herbarium material·······························*P. corneri*6. Hymenial surface brownish in herbarium material··········································*P. gillesii*7. Basidiome infundibuliform or pseudoinfundibuliform················································87. Basidiome flabellate, spathulate, or (pseudo)infundibuliform···································198. Pileus surface tomentose···································································································98. Pileus surface smooth······································································································109. Stipe dark brown in herbarium material, with a hyphal disc··················*P. infundibula*9. Stipe yellowish-to-pale brown in herbarium material, smooth························*P. ursina*10. Hymenial surface tomentose···············································································*P. casiae*10. Hymenial surface smooth·····························································································1111. Hymenial surface with an ashy-gray pruina······························································1211. Hymenial surface without a pruina·············································································1412. Pilei surface with minutely radial wrinkles, without zones···················*P. brasiliensis*12. Pilei surface with darker concentric zones··································································1313. The width of basidiospores up to 2.2 μm··················································*P. fulvonitens*13. The width of basidiospores range from 2.2 μm to 3.2 μm···························*P. ravenelii*14. Stipe lacks a hyphal disc or a conspicuous earth ball at the base·················*P. cristata*14. Stipe with a hyphal disc or a conspicuous earth ball at the base······························1515. Pileocystidia absent, caulocystidia present·································································1615. Pileo- and caulocystidia absent····················································································1716. Basidiospores 3.75–4.75 × 2.5–3.2 μm·····························································*P. bubalina*16. Basidiospores 2.5 × 2 μm··················································································*P. mellissii*17. The length of basidiospores exceed 6 μm························································*P. thozetii*17. The length of basidiospores range from 3 μm to 6 μm···············································1818. Basidiospores up to 2.5 μm broad········································································*P. curta*18. Basidiospores range from 3 μm to 4 μm broad··············································*P. nitidula*19. Pilei surface smooth·······································································································2019. Pilei surface tomentose··································································································3120. Pilei surface dark, blackish brown or black in herbarium material··························2120. Pilei surface warm brown or chestnut in herbarium material··································2221. Pileocystidia present·····················································································*P. ovalispora*21. Pileocystidia absent···························································································*P. moelleri*22. Hymenial surface without a pruina·············································································2322. Hymenial surface with a gray pruina··········································································2623. Stipe tomentose, with a hyphal disc·············································································2423. Stipe without a hyphal disc···························································································2524. Metuloid cystidia present··················································································*P. mellisii*24. Metuloid cystidia absent···················································································*P. elegans*25. Basidiospores 4.2–6.1 × 3.3–4.6 μm···································································*P. tropica*25. Basidiospores 3.75–4.75 × 2.5–3.2 μm·························································*P. glabrescens*26. Stipe without a hyphal disc···························································································2726. Stipe with a hyphal disc·································································································2827. Intermediate hyphae present········································································*P. venustula*27. Intermediate hyphae absent··············································································*P. pusilla*28. Intermediate hyphae present······································································*P. tomentipes*28. Intermediate hyphae abesent························································································2929. Cystidioles thick-walled, up to 52 µm long and 6 µm wide·························*P. parvula*29. Cystidioles absent··········································································································3030. Skeletal hyphae 3–8 μm wide···········································································*P. moselei*30. Skeletal hyphae 3–5 μm wide··········································································*P. bolleana*31. Pilei surface without zones···························································································3231. Pilei surface with zones·································································································3432. Stipe without a hyphal disc···························································*P. xanthopusconcinna*32. Stipe with a hyphal disc·································································································3333. Basidiospores acyanophilous, 3.3–4.3 × 2.6–3 µm·································*P. guangxiensis*33. Basidiospores slightly cyanophilous, 4.6–5.6 × 3–3.8 µm································*P. lactea*34. Pileus surface with a tobacco-brown pruina········································*P. philippinensis*34. Pileus surface without a pruina····················································································3535. Hymenial surface with a pruina···················································································3635. Hymenial surface without a pruina·············································································3936. Stipe with a basal disc····································································································3736. Stipe without a basal disc······························································································3837. Pilei surface light gray, stipe light yellow······················································*P. involuta*37. Pilei surface brown, stipe maroon······························································*P. vespillonea*38. Basidiospores 3.75–5 × 2.5–3.75 µm·······························································*P. petalodes*38. Basidiospores 3–3.75 × 2.2–3 µm·····································································*P. viridans*39. Hymenial surface with radiating ribs····························································*P. replicata*39. Hymenial surface without radiating ribs····································································4040. Basidiospores cyanophilous·········································································*P. densidisca*40. Basidiospores acyanophilous·······················································································4141. Caulocystidia thin- to thick-walled·························································*P. yunnanensis*41. Caulocystidia thick-walled, encrusted with irregular crystals·············*P. subinvoluta*
** **
A key to accepted species of *Cymatoderma*1. Basidiome infundibuliform or pseudoinfundibuliform···············································21. Basidiome flabellate or pseudoinfundibuliform····························································52. Stipe with a hyphal disc······························································*Cymatoderma caperatum*2. Stipe without a hyphal disc·······························································································33. Hymenial surface with radiating ribs···························································*C. africanum*3. Hymenial surface with radial folds··················································································44. Basidiospores elliptical, 8–11 × 4–5.5 μm························································*C. plicatum*4. Basidiospores elliptical to subcylindrical, 4–5.5 × 2–3 μm·······················*C. sclerotioides*5. Pilei surface glabrous····························································································*C. blumei*5. Pilei surface tomentose······································································································66. Basidiospores elliptical, 6.5–9 × 4–5 μm·····························································*C. elegans*6. Basidiospores oval to subglobose, 5–5.8 × 3.8–4.5 μm······································*C. pallens*
** **
A key to accepted species of *Stereopsis*1. Pilei surface tomentose······································································································21. Pilei surface glabrous or with fibers, wrinkles, ridges···················································62. Hymenial surface smooth·································································································32. Hymenial surface tomentose or with radial ridges························································53. Basidiospores subglobose·································································*Stereopsis humphreyi*3. Basidiospores ellipsoid or cylindrical··············································································44. Stipe black············································································································*S. nigripes*4. Stipe tawny to pale brown···············································································*S. straminea*5. Hymenial surface with tiny radial branched ridges······························*S. mussooriensis*5. Hymenial surface tomentose, basidiospores 5.2–6.8 × 5–5.7 μm·············*S. buccinata*6. Pilei surface glabrous·········································································································76. Pilei surface with zones or radial fibers···········································································87. Basidiospores ellipsoid···········································································*S. pseudocupulata*7. Basidiospores subglobose·····················································································*S. albida*8. Cystidia present··················································································································98. Cystidia absent·················································································································119. Cystidia fusiform·························································································*S. yunnanensis*9. Cystidia cylindrical··········································································································1010. Basidia with 2 sterigmata················································································*S. radicans*10. Basidia with 4 sterigmata···················································································*S. raphiae*11. Generative hyphae with clamp connections···················································*S. hiscens*11. Generative hyphae without clamp connections·························································1212. Pilei surface with radial wrinkles or ridges·································································1312. Pilei surface with radial fibers or fibrillose··································································1413. Stipe surface glabrous················································································*S. sparassoides*13. Stipe surface tomentose··············································································*S. cartilaginea*14. Hymenial surface smooth·····························································································1514. Hymenial surface slightly rugulose·············································································1615. Basidiome infundibuliform, stipe surface tomentose··································*S. burtiana*15. Basidiome spathulate or flabelliform··························································*S. malaiensis*16. Basidiospores 4.5–6 × 3–4 µm···············································································*S. reidii*16. Basidiospores 3–4 × 2.2–2.5 µm·······································································*S. vitellina*
** **
A key to accepted species of *Cladoderris*1. Binding hyphae absent·······································································*Cladoderris perennis*1. Binding hyphae branched, 2–2.5 μm·····························································*C. dendritica*

## Figures and Tables

**Table 1:** PCR amplification system.

Reagent	Volume (μL)
Double distilled water	10
2× Taq PCR Mix	12.5
Forward primer	1
Reverse primer	1
DNA	1

**Table 2:** Voucher numbers, geographic origins and GenBank accession numbers used in this study.

Species	Specimen	Locality	ITS	nLSU	References
*Abortiporus biennis*	EL65-03 (GB)	Sweden	JN649325	JN649325	[1]
*A. biennis*	Wei 11890	China, Jiangxi	PQ415519	PQ415507	Unpublished
***Cladoderris perennis***	**Yuan 19310 (T)**	**China, Shenzhen**	**PQ415521**	**PQ415509**	**Present study**
***C. perennis***	**Yuan 19314**	**China, Guangzhou**	**PQ415522**	**PQ415510**	**Present study**
***C. perennis***	**Yuan 19440**	**China, Shenzhen**	**PQ415523**	**PQ415511**	**Present study**
*Clavariadelphus amplus*	HKAS 76577	China	MK705851	MK704443	[43]
*C. amplus*	HKAS 54876 **(T)**	China	MK705857	MK704444	[43]
*C. elongates*	HMAS 260746	China	MK705845	MK704441	[43]
*C. elongates*	MHHNU 9250	China	PV463551	PV490604	[44]
*C. ligula*	KHL 8560 (GB)	Sweden	JN649329	JN649329	[1]
*C. ligula*	MHHNU 10452	China	PV463555	PV490608	[44]
*C. pistillaris*	NAMA 2017-123	USA	MH979250	-	Unpublished
*C. pistillaris*	FLAS-F-60521	USA	MH281842	-	Unpublished
*C. pseudoelongatus*	MHHNU 32323 **(T)**	China	PV463559	PV490617	[44]
*C. pseudoelongatus*	MHHNU 12123	China	PV463560	PV490618	[44]
*C. yunnanensis*	MHHNU 9244	China	PV463561	PV490606	[44]
*C. yunnanensis*	HKAS 54849	China	MK705869	MK704453	[43]
*Clavulicium delectabile*	1391_PREMIX	USA	OQ612508	-	Unpublished
*C. delectabile*	K(M)236345	United Kingdom	MZ159671	-	Unpublished
*C. globosum*	KHL12592	Costa Rica	KC203495	KC203495	[24]
*C. macounii*	Norde’n 37145 (GB)	Sweden	JN649332	JN649332	[1]
*C. macounii*	KHL 12129 (GB)	Sweden	JN649333	JN649333	[1]
*Clavulina castaneipes*	OSC 108705	USA	EU669209	EU669261	Unpublished
*C. castaneipes*	OSC 116725	USA	EU669210	EU669262	Unpublished
*C. cristata*	EL7-00 (GB)	Finland	-	AM259213	[45]
*C. cristata*	RAS323 SV2	USA	OR464380	OR460872	[46]
*C. kunmudlutsa*	TH9206	Guyana	HQ680358	HQ680358	[47]
*C. kunmudlutsa*	TH8932 **(T)**	Guyana	HQ680359	HQ680359	[47]
*C. mahiscolorata*	FCME 27660	Mexico	MH542551	MN049493	[48]
*C. mahiscolorata*	FCME 27662 **(T)**	Mexico	MH542554	MN049496	[48]
*C. parvispora*	FCME 27657	Mexico	MH542549	MN049491	[48]
*C. parvispora*	FCME 27650 **(T)**	Mexico	NR_166245	MN049492	[48]
*C. purpurascens*	MHHNU 9846	China	MK564136	MK564126	[49]
*C. purpurascens*	MHHNU 9848 **(T)**	China	MK564137	MK564127	[49]
*Cymatoderma caperatum*	FLAS-F-61204	USA	MH211809	-	Unpublished
*C. caperatum*	MES-3721	USA	ON383384	-	Unpublished
*C. dendriticum*	CBS 615.73	Sri Lanka	JN649338	JN649338	[1]
*C. dendriticum*	CBS 207.62	Cameroon	JN649339	JN649339	[1]
*C. elegans*	Dai 17511	China	ON417155	-	[50]
*C. elegans*	Yuan 19343	China, Hainan	PQ415520	PQ415508	Unpublished
*C. pallens*	CBS 327.66 **(T)**	Cameroon	JN649342	JN649342	[1]
*Hydnum albomarginatum*	FJAU66574 **(T)**	China	PV329855	PV356813	[51]
*H. albomarginatum*	FJAU66575	China	PV329856	PV356814	[51]
*H. fulvostriatum*	FJAU66566 **(T)**	China	PV329849	PV356807	[51]
*H. fulvostriatum*	FJAU66567	China	PV329850	PV356808	[51]
*H. jussii*	Yuan 14008	China	MW980553	MW979539	[52]
*H. jussii*	Yuan 14009	China	MW980554	MW979540	[52]
*H. pallidocroceum*	Yuan 14023 **(T)**	China	MW980568	MW979554	[52]
*H. pallidocroceum*	Yuan 14017	China	MW980569	MW979555	[52]
*H. repandum*	KHL 8552 (GB)	Sweden	JN649348	JN649348	[1]
*H. repandum*	H 6003710 **(T)**	Finland	NR164553	-	[53]
*H. sphaericum*	Wei 10243 **(T)**	China	MW980563	MW979549	[52]
*H. sphaericum*	Wei 10262	China	MW980565	MW979551	[52]
*Kavinia alboviridis*	KM82737	United Kingdom	GQ981505	-	Unpublished
*K. alboviridis*	EL16-98	Estonia	-	AY463434	[54]
*K. alboviridis*	KM141510	United Kingdom	GQ981506	-	Unpublished
*K. altoandina*	PSL2302 **(T)**	Chile	OP022196	-	[1]
*K. altoandina*	PSL3014	Chile	OP022197	-	[55]
*K. chacoserrana*	Robledo 2516 **(T)**	Argentina	MF377531	PQ453549	Unpublished
*K. himantia*	LL 36041 (GB)	Sweden	-	AY586682	[54]
*K. himantia*	HMJAU59876	China	PX411731	-	Unpublished
*K. himantia*	LE F-355928	Russia	PV017449	-	Unpublished
*Phaeoclavulina abietina*	OSC 134649	USA	JX310378	JX287478	Unpublished
*P. abietina*	EL61-03 (GB)	Sweden	JN649369	JN649369	[1]
*P. bicolor*	MHHNU10702 **(T)**	China	PP809798	PP800475	[56]
*P. bicolor*	MHHNU10703	China	PP809799	PP800476	[56]
*P. echinoflava*	HKAS 45984 **(T)**	China	PP809801	PP800478	[56]
*P. echinoflava*	HKAS 45992	China	PP809800	PP800477	[56]
*P. jilinensis*	MHHNU9149	China	PP809802	PP800479	[56]
*P. jilinensis*	MHHNU9164	China	PP809803	PP800480	[56]
*P. macrospora*	MHHNU9143	China	PP467359	PP493649	Unpublished
*P. macrospora*	MHHNU10399	China	PP467360	PP493650	Unpublished
*P. minutispora*	AMB 18588	Italy	MT055969	MT053246	Unpublished
*P. minutispora*	AMB 18586 **(T)**	Italy	MT055965	MT053243	Unpublished
*Podoscypha bolleana*	CBS 333.66	Central African Republic	JN649354	JN649354	[1]
*P. bolleana*	32032	-	JQ675332	-	Unpublished
*P. brasiliensis*	LR 37812 (O)	Venezuela	JN649355	JN649355	[1]
*P. bubalina*	17500	-	JQ675311	-	Unpublished
***P. casiae***	**Yuan 19306 (T)**	**China, Shenzhen**	**PQ415526**	**PQ415513**	**Present study**
***P. casiae***	**Dai 7499**	**China, Shenzhen**	**PQ415527**	**PQ415514**	**Present study**
***P. casiae***	**Yuan 19320**	**China, Shenzhen**	**PQ415528**	**PQ415515**	**Present study**
*P. cristata*	8667	-	JQ675320	-	Unpublished
*P. densidisca*	GXU 5784 **(T)**	China, Guangxi	OR347558	OR347508	[13]
*P. densidisca*	GXU 7524	China, Guangxi	OR347571	OR347572	[13]
*P. disseminata*	DMC 232	-	JQ675326	-	Unpublished
*P. elegans*	CBS 426.51	Argentina	JN649356	JN649356	[1]
*P. fulvonitens*	17483	-	JQ675315	-	Unpublished
*P. fulvonitens*	18332	-	JQ675316	-	Unpublished
*P. gillesii*	GXU 2176	China	MG356710	MG356793	[12]
*P. guangxiensis*	GXU 5849 **(T)**	China, Guangxi	OR347573	OR347564	[13]
*P. guangxiensis*	GXU 6031	China, Guangxi	OR347565	OR347566	[13]
*P. infundibula*	GXU 7837 **(T)**	China, Guangxi	OR340036	OR340137	[13]
*P. infundibula*	GXU 8829	China, Guangxi	OR342113	OR342114	[13]
*P. involuta*	E. Larsson (GB)	Thailand	JN649357	JN649357	[1]
*P. involuta*	CBS 113.74	Central African Republic	JN649358	JN649358	[1]
*P. lactea*	GXU 5952 **(T)**	China, Guangxi	OR347570	OR347567	[13]
*P. lactea*	GXU 7612	China, Guangxi	OR347569	OR347569	[13]
*P. mellissii*	LR 41658 (O)	Jamaica	JN649359	JN649359	[1]
*P. multizonata*	Jahn 751012 (GB)	Germany	JN649360	JN649360	[1]
*P. multizonata*	3005	Germany	JN710581	JN710581	[57]
*P. nitidula*	8724	-	JQ675321	-	Unpublished
*P. parvula*	32055	-	JQ675338	-	Unpublished
*P. parvula*	DMC 226	-	JQ675328	-	Unpublished
*P. petalodes*	Yuan 1634	China	PQ415525	-	Unpublished
*P. petalodes* subsp. *rosulata*	CBS 659.84	Pakistan	JN649362	JN649362	[1]
*P. petalodes* subsp. *rosulata*	CBS 332.66	Pakistan	JN649363	JN649363	[1]
*P. petalodes* subsp. *cystidiata*	140721-15 **(T)**	China: Yunnan	OQ305833	OQ305828	[15]
*P. petalodes* subsp. *cystidiata*	140721-14	China: Yunnan	OQ305832	OQ305827	[15]
*P. ravenelii*	CBS 664.84	USA	JN649364	JN649364	[1]
*P. sp.*	LR43794 (O)	Costa Rica	JN649365	JN649365	[1]
*P. tropica*	140719-19 **(T)**	China: Yunnan	OQ305834	OQ305830	[15]
*P. tropica*	Yuan 19438	China, Jiangxi	PQ415524	PQ415512	Unpublished
*P. venustula*	LR40821 (O)	Venezuela	JN649366	JN649366	[1]
*P. venustula*	CBS 656.84	French Guiana	JN649367	JN649367	[1]
*P. vespillonea*	CBS 111.74	-	JN649368	JN649368	[1]
*P. vespillonea*	CBS 111.74	-	MH860836	-	[58]
*P. yunnanensis*	CLZhao 3963	China	MK298400	MK298404	[10]
*P. yunnanensis*	CLZhao 4035 **(T)**	China	MK298403	MK298407	[10]
*Protodontia piceicola*	KHL 11763 (GB)	Sweden	DQ873660	DQ873660	[59]
*Sistotrema brinkmannii*	NH 11412 (GB)	Turkey	AF506473	AF506473	[60]
*S. brinkmannii*	KHL14078 (GB)	Sweden	KF218967	KF218967	[61]
*S. confluens*	MV-1754	Sweden	PQ653462	PQ653462	Unpublished
*S. confluens*	RAS950	USA	PQ050701	PQ050699	[62]
*S. flavorhizomorphae*	TUMH 64409 **(T)**	Japan	NR_178118	NG_153858	[63]
*S. flavorhizomorphae*	TUMH 64408	Japan	LC642048	LC642066	[63]
*S. luteoviride*	HK23176 (H) **(T)**	Finland	KF218963	KF218963	[61]
*S. sinense*	CLZhao 24876 **(T)**	China	PQ758748	PQ758756	[64]
*S. subconfluens*	Dai 12577 **(T)**	China	-	JX076810	[65]
*S. subconfluens*	Dai 12578	China	-	JX076811	[65]
*S. yunnanense*	CLZhao 7357 **(T)**	China	ON817194	ON810362	[66]
*S. yunnanense*	CLZhao 7396	China	ON817195	ON810363	[66]
*Steccherinum ochraceum*	KHL 11902 (GB)	Sweden	JQ031130	JQ031130	[1]
***Stereopsis buccinata***	**Yuan 18472 (T)**	**China, Guangzhou**	**PV190982**	**PV174382**	**Present study**
*S. globosa*	KHL 11228 (GB)	Costa Rica	JN649330	JN649330	[1]
*S. globosa*	KHL 12592 (GB)	Costa Rica	JN649331	JN649331	[1]
*S. radicans*	PR 5760 **(T)**	Puerto Rico	JN649370	JN649370	[1]
*S. radicans*	TB 8943 (CORT)	Venezuela	JN649372	JN649372	[1]
*S. yunnanensis*	CLZhao 3767 **(T)**	China, Yunnan	NR198372	NG243203	[25]
*S. yunnanensis*	Yuan 19365	China, Shenzhen	PV190983	PV174383	Unpublished

New species are in bold, and type specimens are indicated by (T). Specimen Deposition: (GB) = Herbarium, University of Gothenburg (Sweden); (O) = Oslo Herbarium, Natural History Museum, University of Oslo (Norway).

## Data Availability

The sequences from the present study were submitted to the NCBI website (https://www.ncbi.nlm.nih.gov/, accessed on 29 June 2025), and the accession numbers are listed in Table 2.

## Supplementary Materials

The following supporting information can be downloaded at: https://www.mdpi.com/article/10.3390/jof12060400/s1, Supplementary Material S1: The bPTP species delimitation analysis of the genera *Podoscypha*, *Cymatoderma*, and *Cladoderris*; Supplementary Material S2: The bPTP species delimitation analysis of the genus *Stereopsis*; Supplementary Material S3: Genetic distance of the genera *Podoscypha*, *Cymatoderma*, and *Cladoderris* based on ITS + nLSU.
